# Hyunganol II Exerts Antiadipogenic Properties via MAPK-Mediated Suppression of PPAR*γ* Expression in Human Bone Marrow-Derived Mesenchymal Stromal Cells

**DOI:** 10.1155/2022/4252917

**Published:** 2022-10-17

**Authors:** Jung Hwan Oh, Fatih Karadeniz, Youngwan Seo, Chang-Suk Kong

**Affiliations:** ^1^Marine Biotechnology Center for Pharmaceuticals and Foods, College of Medical and Life Sciences, Silla University, Busan 46958, Republic of Korea; ^2^Nutritional Education, Graduate School of Education, Silla University, Busan 46958, Republic of Korea; ^3^Division of Convergence on Marine Science, College of Ocean Science and Technology, Korea Maritime and Ocean University, Busan 49112, Republic of Korea; ^4^Department of Food and Nutrition, College of Medical and Life Sciences, Silla University, Busan 46958, Republic of Korea

## Abstract

Bone marrow adiposity has been associated with several metabolic syndromes such as diabetes and osteoporosis. Imbalance in adipogenic and osteoblastogenic differentiation of human bone marrow mesenchymal stromal cells (hBM-MSCs) was suggested to be the cause of elevated bone marrow adiposity. There are several drugs, of both natural and synthetic origin, to treat bone loss. In this study, as a part of a recent trend to discover natural products with more biocompatibility and fewer side effects to treat bone loss, the effect of hyunganol II (HNG), a coumarin isolated from *Corydalis heterocarpa*, on hBM-MSC adipogenesis was investigated. Cells treated with HNG showed decreased lipid accumulation indicating a diminished adipocyte phenotype. Treatment with HNG also suppressed the mRNA and protein expressions of PPAR*γ*, C/EBP*α*, and SREBP1c, and three adipogenic marker genes. Further analysis of MAPK signaling pathway exhibited that HNG treatment elevated ERK activation and suppressed the JNK-mediated cFos and cJun phosphorylation, which inhibits PPAR*γ* transcriptional activity. Taken together, HNG treatment was shown to inhibit adipogenesis via suppressed PPAR*γ* expression as a result of altered MAPK signaling. Therefore, it was suggested that HNG might prevent bone marrow adiposity by inhibiting hBM-MSC adipogenesis and can be utilized as a drug or nutraceutical with beneficial effects on bone. Thus, further studies should be conducted to analyze its effect in vivo.

## 1. Introduction

Bone marrow in humans hosts nonhematopoietic multipotent stem cells that can be induced to differentiate into different types of cell lineages [1]. These cell lineages include osteoblasts, chondrocytes, and adipocytes, all of which play crucial roles in bone formation and homeostasis as well as energy metabolism [2]. In terms of the latter, bone marrow adipose tissue is the main regulatory factor consisting of adipocytes arising from human bone marrow mesenchymal stromal cells (hBM-MSCs). Metabolic syndromes such as osteoporosis, diabetes, and obesity have been known to present high risk factors for bone-related disorders mainly due to bone loss [3, 4]. Studies showed that these diseases are also in close relation with bone marrow adiposity and an increase in bone marrow fat accumulation was observed in the progression of these metabolic syndromes [5]. Griffith [6] reported that analyses of osteoporotic patients revelated reduced bone formation coupled with elevated bone marrow adipose tissue volume. Similar results were obtained from studies on patients suffering from diabetes and obesity [7, 8]. Overall, it was suggested that bone marrow adiposity is a common complication and risk factor for bone-related diseases, especially osteoporosis.

Increase in bone marrow adiposity is considered a result of overstimulated differentiation of hBM-MSCs into adipocytes rather than osteoblasts [9]. Studies showed that enhanced hBM-MSC adipogenesis is associated with lower bone formation and therefore critical bone loss [5, 10, 11]. There are several drugs on the market, both natural origin such as phytoestrogens [12] or synthetic origin such as bisphosphonates [13] that target bone loss to prevent and treat osteoporosis. The mechanism of action of the drugs intended to be used against bone disorders are mainly either prevention of bone resorption to keep bone levels intact [14] or induce osteoblast differentiation to increase bone cells [15]. However, considering the role of bone marrow adiposity in metabolic syndrome, novel approaches such as prevention of bone marrow adiposity by targeting hBM-MSC differentiation are needed.

Using natural sources to isolate and characterize bioactive molecules that may act as lead compounds for drug discovery is a traditional and common method as such more than 60% of the approved drugs are derived from natural products between the years 1981 and 2010 [16]. In this context, several bioactive molecules were reported with adipogenesis inhibitory effect on hBM-MSCs, while some of them also enhanced osteoblastogenesis as well [17–19]. *Corydalis heterocarpa* is a halophyte with reported MMP inhibitory, antiphotoaging and anti-inflammatory bioactivities [20]. Reports showed that one of the main active constituents of *C. heterocarpa* are coumarins and hyunganol II (HNG) is such a coumarin found in *C. heterocarpa* [21]. However, the literature lacks any study on HNG apart from a report by Kim et al. who showed that among other coumarins, HNG showed a slight antioxidant and anti-inflammatory effect. The current study aimed to investigate the potential effect of HNG on the adipogenic differentiation of hBM-MSCs in vitro, to provide insights on its potential utilization as an antiadipogenic compound with beneficial effects on bone marrow adiposity.

## 2. Materials and Methods

### 2.1. Isolation and Characterization of HNG

Whole *C. heterocarpa* plants were extracted, and HNG was isolated among other coumarin derivatives as described earlier [21]. In the present study, HNG was dissolved in distilled water to be used in the cell-based experiments.

### 2.2. Cell Maintenance and Differentiation

Human bone marrow-derived mesenchymal stromal cells were procured from PromoCell (hBM-MSC, C-12974). Cells were cultured in flat bottom transparent 6-well plates with 4 × 10^4^ cells/well seeding density and fed Mesenchymal Stem Cell Growth Medium 2 (C-28009, PromoCell) unless otherwise noted. Cultured cells were kept in incubators with 37°C temperature and 5% CO_2_ between experiments. Adipogenic differentiation of hBM-MSCs were induced in cells that reached confluency by swapping cell culture medium with Mesenchymal Stem Cell Adipogenic Differentiation Medium 2 (C-28016, PromoCell). Induced cells were cultured for 14 days with periodical medium changes (every third day) for cells to reach a mature adipocyte state. For treatment groups, HNG was present in the initial differentiation medium only and subsequent medium changes did not contain HNG. A blank group without differentiation inducement and HNG treatment, and a control group with adipogenic differentiation but not HNG were cultured.

### 2.3. Oil Red O Staining of Intracellular Lipid Droplets

Adipogenic character of differentiating hBM-MSCs was screened by staining intracellular lipid droplets, which are characteristic of mature adipocytes via Oil Red O staining. Cells were cultured and induced for adipogenesis as described earlier. At day 14 of adipogenesis, the differentiation medium was removed and cells were gently washed with PBS without disturbing the adipocyte layer. Next, cells were fixed to the wells by adding 1 ml 10% fresh formaldehyde (in PBS, v/v) to each well. After keeping the plates for 1 h at room temperature, the wells were aspirated and cells were stained by 1 ml 0.5% Oil Red O solution (m/v, in 60% isopropanol and 40% water). Plates were kept at room temperature for 1 h, after which the staining solution was removed and the wells were air-dried. Following staining, intracellular lipid droplets were observed under light microscope and red spots (Olympus, Tokyo, Japan). The quantification of intracellular lipid droplets was carried out by measuring the absorbance values of stain from each well. Oil Red O stain was eluted from each well by adding 2 ml 100% isopropyl alcohol. Then, quantification was carried out by measuring the absorbance of the wells at 500 nm using a microplate reader (MultiSkan GO, Tecan, Austria). Lipid droplet amount was calculated as a relative percentage of the differentiated but not treated control group.

### 2.4. Analysis of mRNA Levels by Reverse Transcriptase Quantitative Polymerase Chain Reaction (RT-qPCR)

The hBM-MSCs were cultured and differentiated as described earlier. The mRNA expression levels of adipogenic factors in differentiating hBM-MSCs were analyzed by RT-qPCR. Total RNA was isolated from hBM-MSCs at day 14 of differentiation using a commercial RNA extraction kit (AccuPrep® Universal Bioneer, Daejeon, Korea) following the enclosed direction. RNase-free DNase I (Thermo Fisher Scientific, Rockford, IL, USA) treated total RNA was then converted to cDNA using CellScript All-in-One cDNA synthesis Master Mix (CellSafe, Yongin, Korea) following the manufacturer's protocol. Target specific cDNA amplification via real-time PCR was carried out in a Dice Real Time System (TP800, Takara Bio, Ohtsu, Japan) with the help of Luna Universal qPCR mix (New England Biolabs, Ipswich, MA, USA) and gene-specific forward and reverse primers given in detail earlier [22]. *β*-actin was used as a reference gene for the calculation of the relative target gene amount.

### 2.5. Western Blotting

The hBM-MSCs were cultured, treated, and induced to differentiate as described earlier. To analyze the adipogenic marker protein levels, the total protein was isolated from cells by obtaining cell lysates via addition of 1 ml RIPA buffer (R0278; Sigma-Aldrich, St. Loius, MI, USA) supplemented with protease and phosphatase inhibitor cocktail (78446; Thermo Fisher Scientific, Waltham, MA, USA) to each well after media removal and subsequent washing of cells in PBS. Cell lysates were then centrifuged at 12000 rpm for 10 min and the supernatants were used for Western blotting. Protein concentration was calculated using BCA protein assay kit (Thermo Fisher Scientific) and the same amount of protein from each treatment group was loaded on a 10% SDS-PAGE gel. Following SDS-PAGE, proteins were transferred to nitrocellulose membranes. Blotted membranes were then blocked in 5% skim milk for 4 h at room temperature and hybridized with primary antibodies against PPAR*γ* (^#^2443; Cell Signaling Technology, Danvers, MA, USA), C/EBP*α* (^#^2295; Cell Signaling Technology), SREBP1c (ab3259; Abcam, Cambridge, MA, USA), p38 (^#^8690; Cell Signaling Technology), phosphorylated (p-) p38 (^#^4511; Cell Signaling Technology), JNK (LF-PA0047; Thermo Fisher Scientific), p-JNK (^#^9255; Cell Signaling Technology), ERK (^#^4695; Cell Signaling Technology), p-ERK (^#^4370; Cell Signaling Technology), cFos (sc-7202; Santa Cruz Biotechnology, Santa Cruz, CA, USA), p-c-Fos (^#^5348s; Cell Signaling Technology), cJun (sc-74543; Santa Cruz Biotechnology), p-c-Jun (sc-822; Santa Cruz Biotechnology), *β*-actin (sc-47778; Santa Cruz Biotechnology), and lamin B1 (sc-374015; Santa Cruz Biotechnology) overnight at 4°C. Hybridized membranes were incubated with horse radish peroxidase conjugated anti-mouse and anti-rabbit antibodies for 1 h at room temperature. Protein bands on membranes were visualized with a commercial chemiluminescence kit (Amersham ECL detection kit, GE Healthcare, Chicago, IL, USA), and the images were taken with Davinch-Chemi imager (CAS-400M, Davinch-K, Seoul, Korea).

### 2.6. Immunofluorescence Staining

Intracellular level of the adipogenic transcription factor, PPAR*γ*, was also observed by immunofluorescence staining. The hBM-MSCs were cultured on glass coverslips, placed into 6-well plate wells, and induced to differentiate as described earlier. At day 7 of differentiation, cells were fixed on glass coverslips and loaded with anti-PPAR*γ* antibody attached with ALexa Fluor 488 Green (A-11008; Invitrogen, Carlsbad, CA, USA) and ProLong Gold Antifade Reagent with 4′,6-diamidino-2-phenylindole (DAPI) (^#^8961; Cell Signaling Technology) for the detection of cell nuclei by commercial Immunofluorescence Application Solutions Kit (^#^12727; Cell Signaling Technology) following manufacturer's protocol.

### 2.7. Statistical Analysis

All numerical data of the current study were given as a mean of three different experiments ± SE. Any statistical difference between treatment groups was defined at *p* < 0.05 comparing the results of one-way ANOVA with Duncan's multiple range test as post hoc. Each treatment group was denominated with a letter, and identical letters indicate no statistical difference.

## 3. Results and Discussion

### 3.1. Effect of HNG on Adipogenic Differentiation of hBM-MSCs

**Figure 1:**
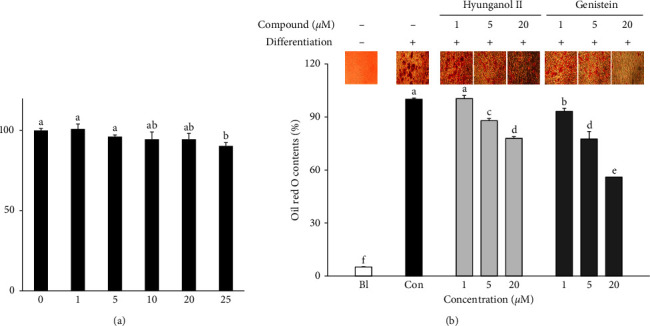
Effect of HNG on the intracellular lipid accumulation of differentiating hBM-MSCs. (a) Cytotoxicity of HNG in hBM-MSCs was evaluated with MTT assay. (b) Intracellular lipid droplets were stained by Oil Red O at day 14 of differentiation. Lipid droplet quantification was carried out by measuring the stain in the cells and given as a relative percentage of differentiated untreated control. Bl: nondifferentiated untreated cells, Con: differentiated untreated cells. ^a–d^Bars with identical letters indicate no statistical significance (*p* < 0.05), while different letters indicate otherwise.

Prior to analyzing the effect of HNG on adipogenic differentiation of hBM-MSCs, any possible cytotoxicity induced by HNG treatment was analyzed by MTT. Results showed that HNG treatment for 3 days did not result in any significant toxicity in hBM-MSCs until 20 *μ*M (Figure 1(a)). Therefore, the following assays were carried out using HNG in this range.

Initially, the ability of hBM-MSCs to differentiate into adipocytes was confirmed by Oil Red O staining of the intracellular lipid accumulation as a mature adipocyte phenotype. The hBM-MSCs without any treatment differentiated into adipocytes and expressed significant amounts of lipid droplets compared to nondifferentiated nontreated group (Figure 1(b)). There were significant changes in lipid droplet accumulation of differentiating hBM-MSCs treated with HNG and genistein. Genistein is a natural product, a phytoestrogen mainly found in legumes.

It is chemically similar to estrogens [23]. Hence, it has been reported to exert beneficial effects on postmenopausal osteoporosis due to its bone-protective effects [24]. It has been used as a comparable positive control in the current study, to evaluate the effects of HNG on MSC adipogenic differentiation and provide insights on its potential utilization as a bone-preventive natural product. Cells treated with HNG during the initial adipogenesis inducement showed dose-dependently decreased lipid accumulation, hinting at a hindered adipogenesis and adipocyte phenotype, comparable to genistein. Apart from the amount, HNG-treated cells expressed lipid droplets smaller in size compared to nontreated cells.

**Figure 2:**
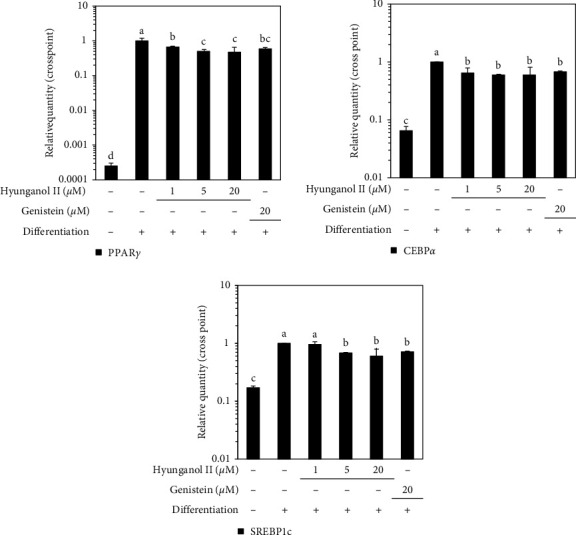
Effect of HNG on the mRNA expression of adipogenic marker genes, PPAR*γ*, C/EBP*α*, and SREBP1c in differentiating hBM-MSCs analyzed by RT-qPCR. Expression levels of marker gene mRNAs were analyzed on day 14 of differentiation. Changes in mRNA expression were given as relative fold changes compared with differentiated untreated control. ^a–d^Bars with identical letters indicate no statistical significance (*p* < 0.05), while different letters indicate otherwise.

**Figure 3:**
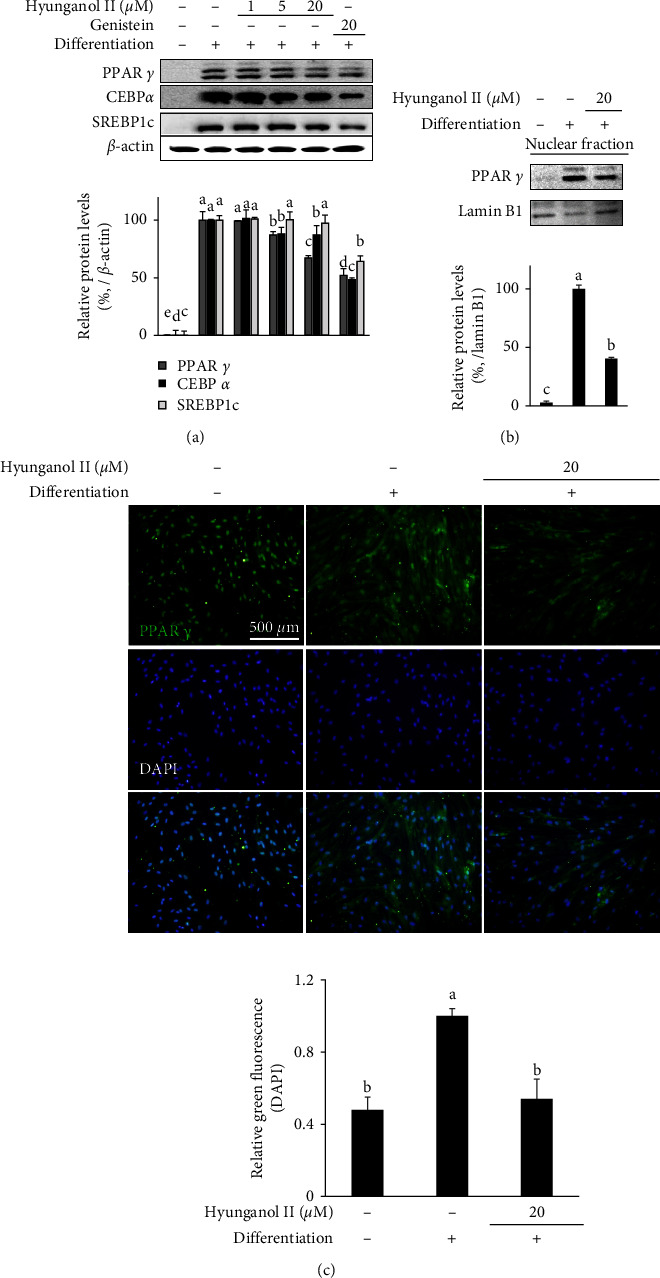
Effect of HNG on the protein expression of adipogenic marker genes, PPAR*γ*, C/EBP*α*, and SREBP1c in differentiating hBM-MSCs. (a) The whole cell and (b) nuclear protein levels were analyzed with Western blotting. *β*-actin and lamin B1 were used as internal controls for whole cell and nuclear protein levels, respectively. (c) Intracellular PPAR*γ* protein was stained with immunofluorescence staining (green) and DAPI nuclear staining (blue) was used as viable cell control. ^a–e^Bars with identical letters indicate no statistical significance (*p* < 0.05), while different letters indicate otherwise.

The mRNA expression of three marker genes for adipogenic differentiation was investigated to elucidate the effect of HNG on intracellular signaling of hBM-MSC adipogenesis. The expressions of PPAR*γ*, C/EBP*α*, and SREBP1c were analyzed by RT-qPCR. The expression of all three adipogenic marker genes was inhibited by HNG treatment (Figure 2). Inhibition of these adipogenesis inducing marker genes showed that HNG treatment suppressed the adipocyte phenotype of differentiating hBM-MSCs via inhibition of adipogenesis. Inhibitory effect of HNG on PPAR*γ* pathway was further confirmed by Western blotting. The protein expression levels of three adipogenic marker genes were inhibited by HNG treatment (Figure 3(a)). Aside from this, the nuclear fraction of differentiated hBM-MSCs showed that 20 *μ*M HNG treatment significantly decreased the nuclear presence of PPAR*γ* protein, suggesting the decrease in transcriptional activity and consequent decrease in adipogenic characteristics (Figure 3(b)). This decrease in PPAR*γ* expression and nuclear translocation was also analyzed.

By immunofluorescence staining of intracellular PPAR*γ* protein, images showed that the adipogenesis inducement-related increase in PPAR*γ* expression was significantly reduced with 20 *μ*M HNG treatment (Figure 3(c)). Overall, it was shown that HNG treatment inhibited both mRNA and protein expression of PPAR*γ* and therefore, suggestively inhibited adipogenic differentiation in hBM-MSCs.

In all steps, the inhibitory effect of HNG was comparable to that of genistein which like other estrogen-like compounds possess side effects [25]. Although there are no data suggesting that HNG does not possess similar side effects, overall results indicated that its effect on bone marrow adipogenesis was on par with genistein.

### 3.2. Effect of HNG on MAPK Signaling in Differentiating hBM-MSCs

To investigate and elucidate the possible action mechanism for the antiadipogenic effect of HNG in hBM-MSCs, the activation of MAPK signaling was analyzed. The MAPK signaling pathway plays roles in all steps of adipogenic differentiation [26]. The activation of JNK.

**Figure 4:**
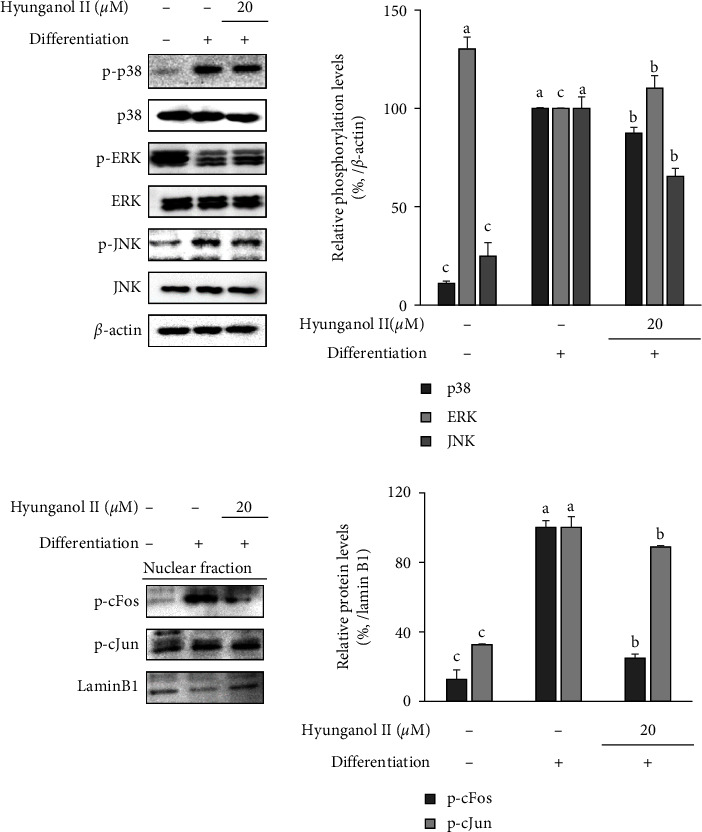
Effect of HNG on the activation of MAPK signaling in differentiating hBM-MSCs. The whole cell protein levels of phosphorylated (p-) and total protein levels of p38, ERK, and JNK, and nuclear protein levels of p-cFos and p-cJun was analyzed by Western blotting. *β*-actin and lamin B1 were used as internal controls for whole cell and nuclear protein levels, respectively. ^a–e^Bars with identical letters indicate no statistical significance (*p* < 0.05), while different letters indicate otherwise.

MAPK is crucial for the transcriptional activity of AP-1, a dimer formed by phosphorylated Fos and Jun proteins. AP-1 transcriptional activity further stimulates adipogenic differentiation and maturity [27]. On the other hand, ERK and p38 MAPKs are pivotal for cell clonal proliferation and subsequent differentiation processes, which are essential for adipogenesis [26–29]. While activation of JNK and related cFos and cJun proteins stimulate adipogenesis, activation of ERK might play roles in the downregulation of adipogenic gene expression. Western blot analysis showed that hBM-MSCs that were induced to differentiate, expressed elevated JNK, and p38 activation as increased phosphorylation along with suppressed ERK phosphorylation compared to non-differentiated nontreated group (Figure 4). However, treatment with 20 *μ*M HNG reversed the adipogenesis-mediated changes in MAPK activation. Although cells treated with HNG did not show any significant changes in p38 activation, HNG treatment increased phosphorylated ERK levels and decreased the phosphorylated JNK. The effect of HNG on JNK activation was further confirmed by the nuclear levels of phosphorylated cFos and cJun. The suppressive effect of HNG translated into decreased phosphorylated cFos levels in the nucleus. This was suggested to lead to inhibited AP-1 activity, which was reported to lead to stalled adipogenesis.

ERK phosphorylation is the upstream step for the phosphorylation of PPAR*γ*, which leads to suspended transcriptional activity and consequent inhibition of adipogenesis [28]. Therefore, the effect of HNG on increasing ERK phosphorylation was suggested to be the reason behind its inhibitory effect on PPAR*γ* pathway. Increased ERK activation during adipogenesis suppresses the PPAR-mediatedadipo-inducement.

Increased bone marrow adiposity has been observed in patients with obesity, diabetes, and osteoporosis [3, 4]. It has been shown to be one of the causes of fragile bone and chronic bone loss [30]. Studies showed that bone marrow adiposity increases in these conditions due to an imbalance in bone marrow stromal cell differentiation where adipogenesis dominates over osteoblastogenesis resulting in adiposity and lack of bone formation [30, 31]. Therefore, focusing on finding molecules that target this imbalance and alter the differentiation of bone marrow cells might have clinical uses.

## 4. Conclusions

Current results indicated that the HNG treatment inhibits the adipogenic differentiation of hBM-MSCs, which might exert beneficial effects on bone marrow adiposity observed in osteoporotic conditions. Despite the lack of in vivo confirmation, the present study contributes towards the utilization of HNG as a natural product with potential antiosteoporotic properties. Taken together, the present data suggests that further studies focusing on the antiosteoporotic acitivities of HNG would yield valuable results which could be utilized to develop novel natural products against bone disorders. In conclusion, HNG was shown to inhibit adipogenesis in hBM-MSCs via MAPK-mediated inhibition of PPAR*γ* pathway and utilization of HNG as a novel nutraceutical is possible with further studies to confirm its effect.

## Data Availability

All the data used to support the findings of this study are available from the corresponding author upon reasonable request.
