# Supplementary material for: Hyunganol II Exerts Antiadipogenic Properties via MAPK-Mediated Suppression of PPARγ Expression in Human Bone Marrow-Derived Mesenchymal Stromal Cells
Source: Evid Based Complement Alternat Med. 2022 Oct 17;2022:4252917. doi: 10.1155/2022/4252917 (PMC9592193; doi:10.1155/2022/4252917)

Figure 3A - PPARγ

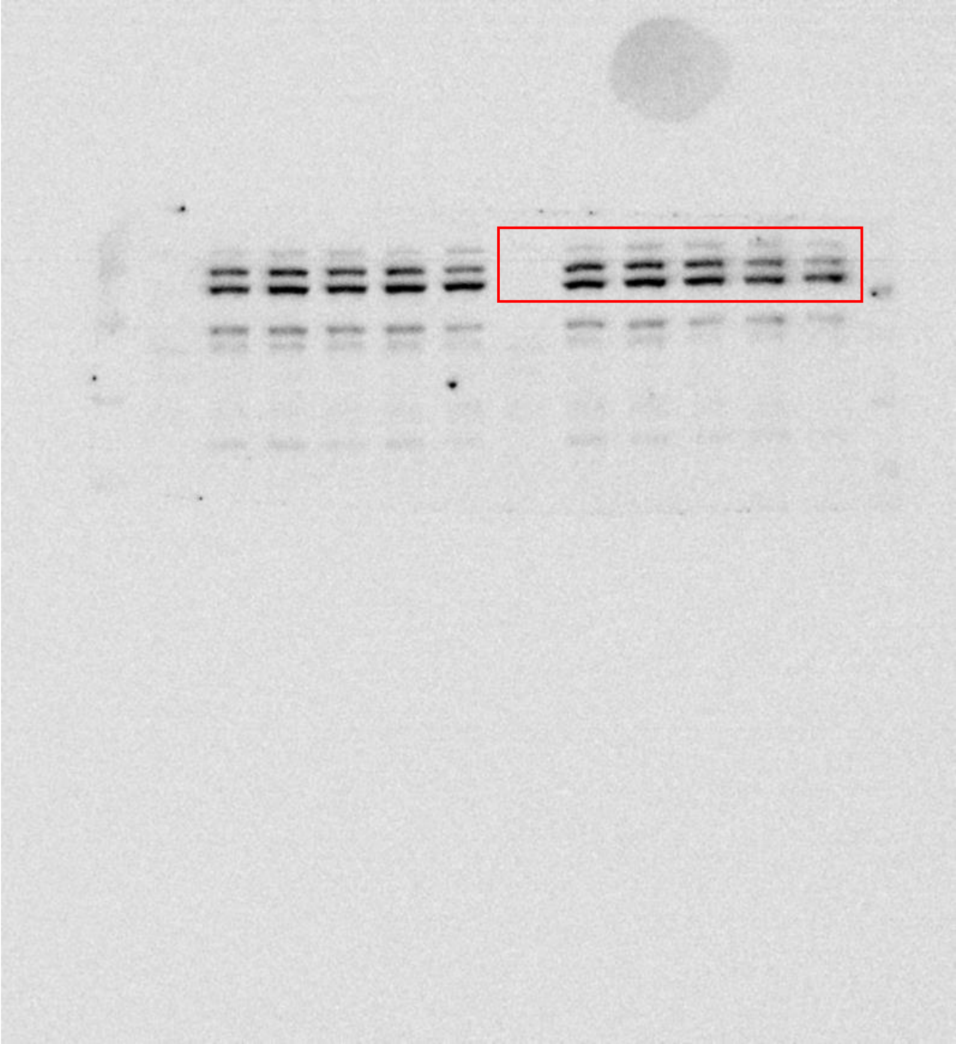

Figure 3A – C/EBPα

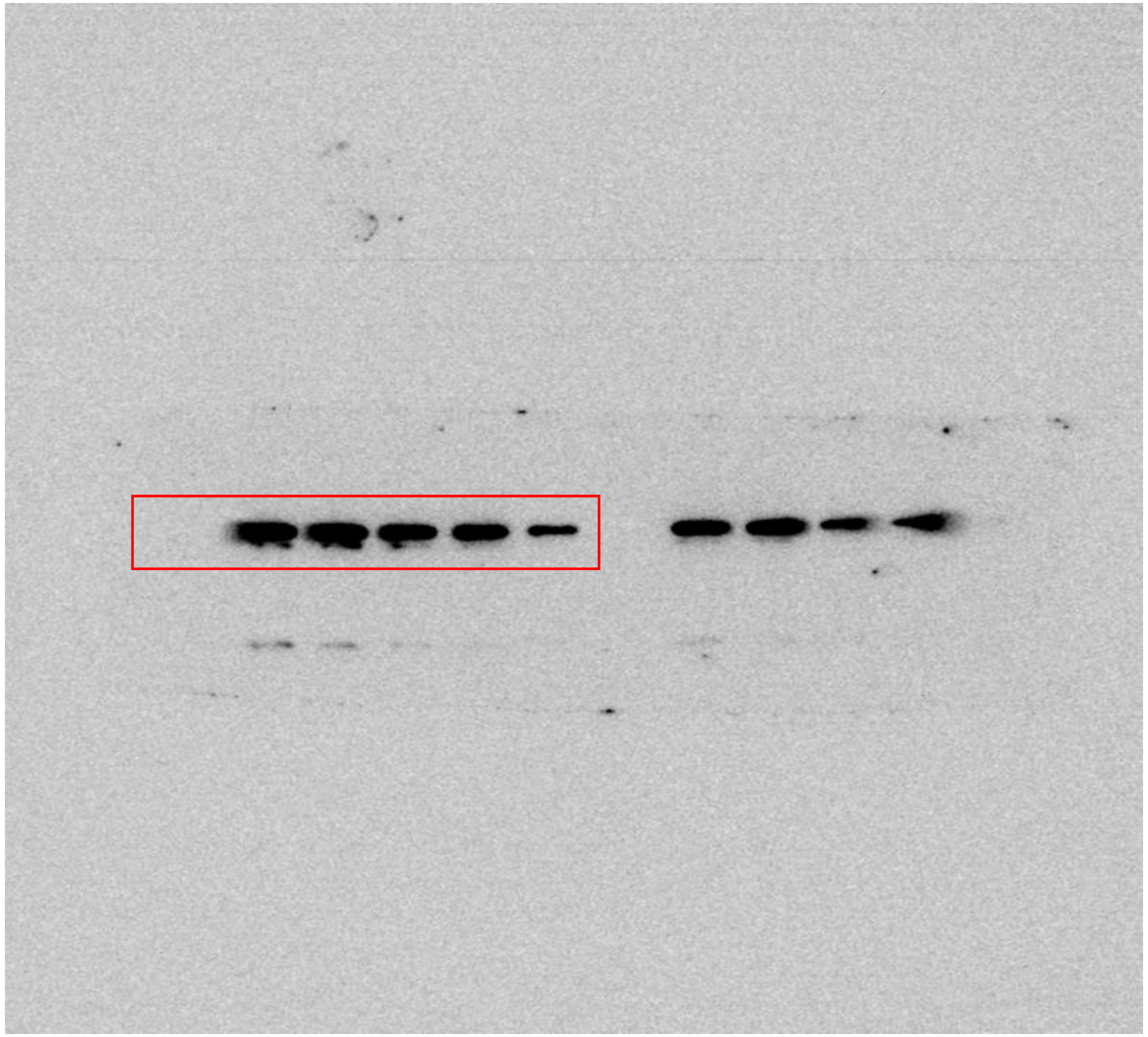

Figure 3A – SREBP1c

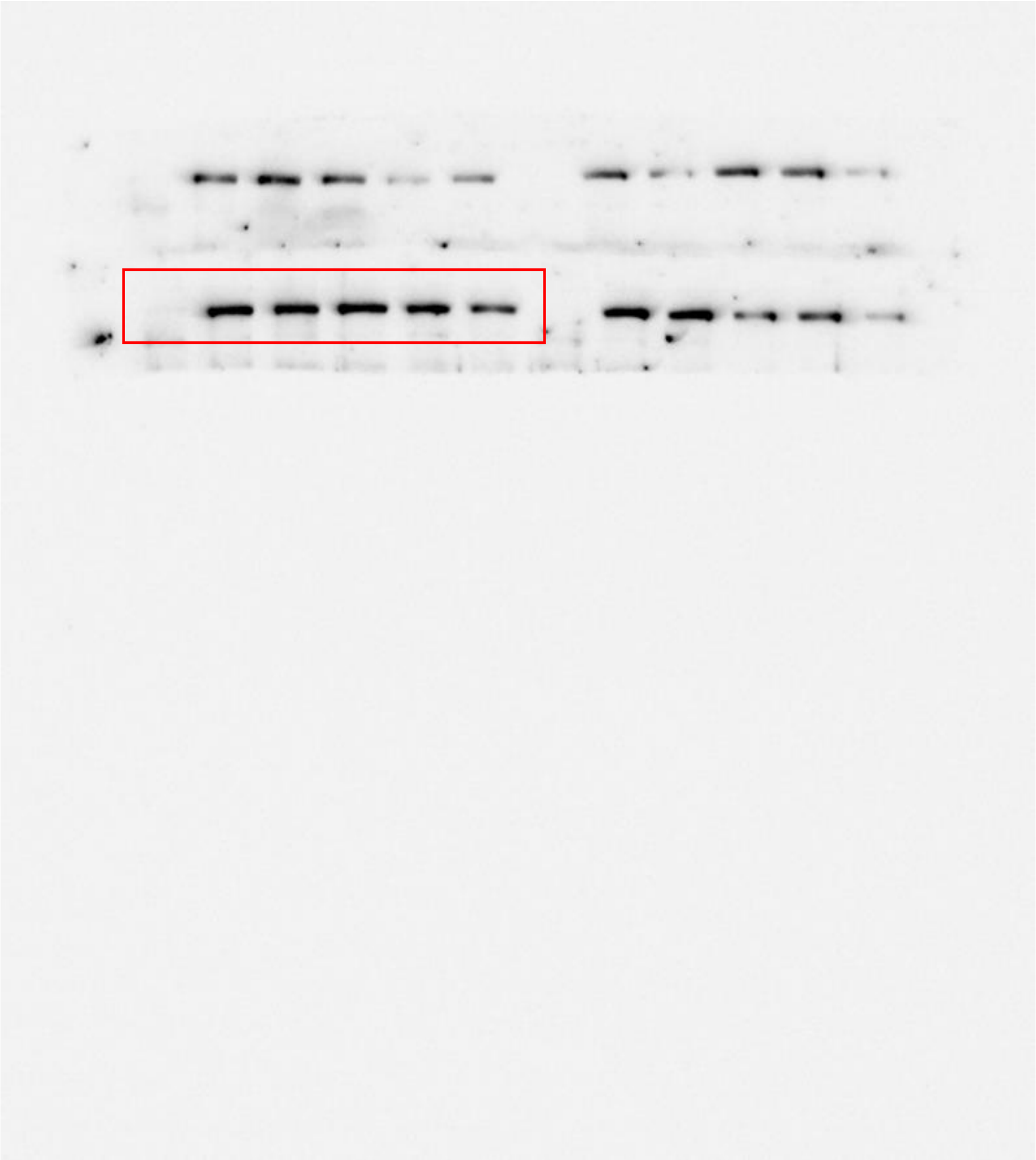

Figure 3A – β-actin

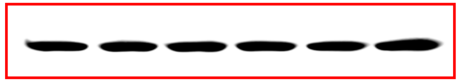

Figure 3B – PPARγ

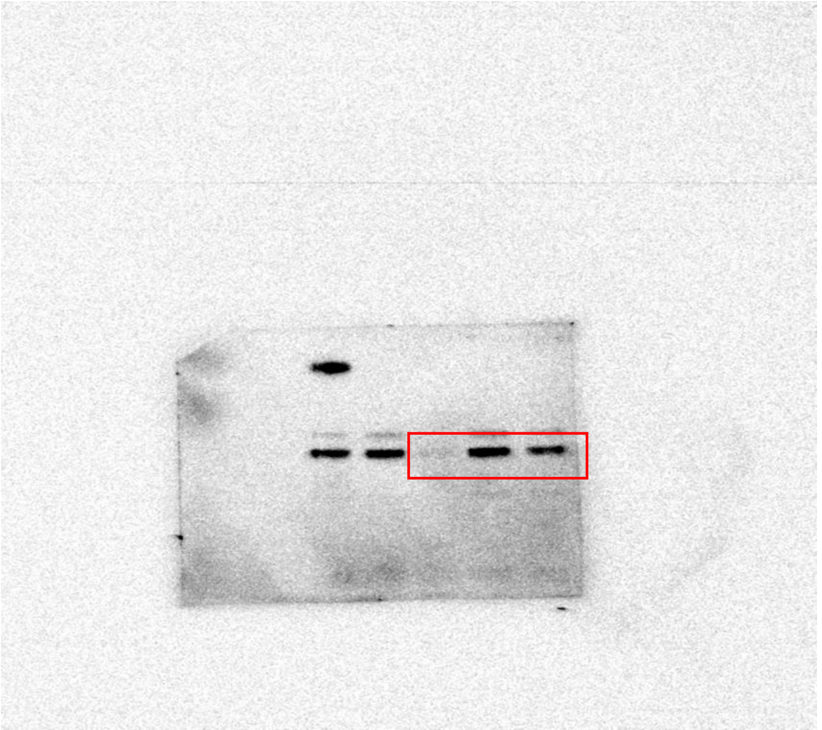

Figure 3B – lamin B1

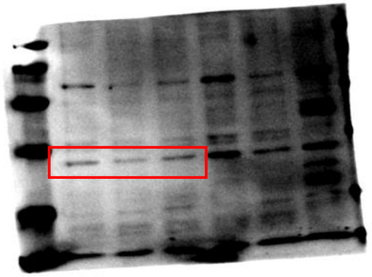

Figure 4 – p-p38

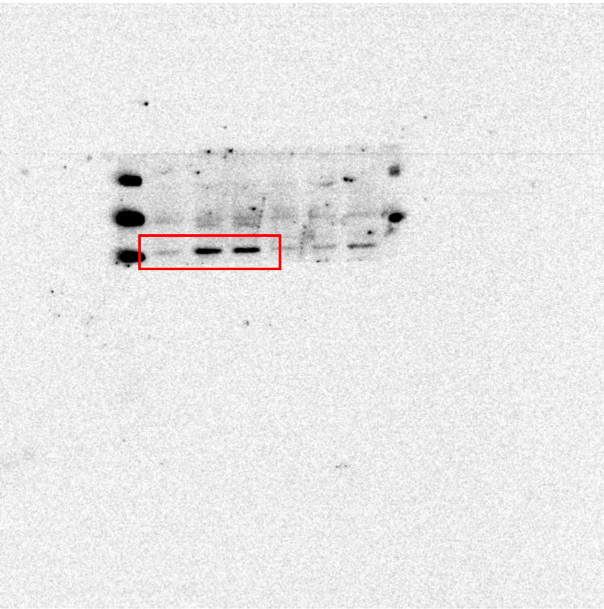

Figure 4 – p-ERK

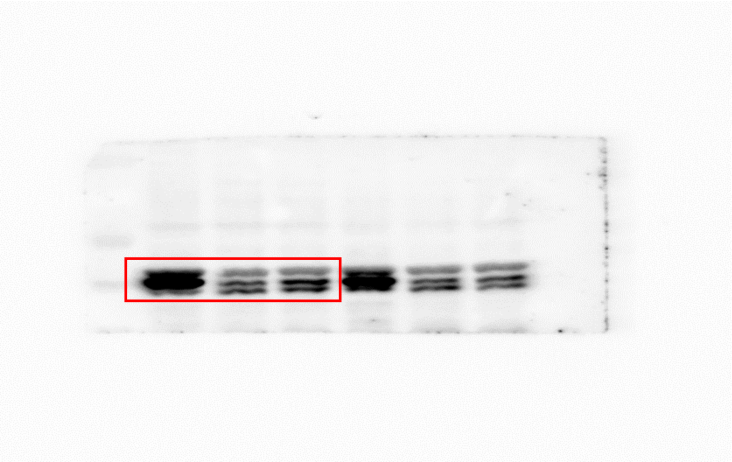

Figure 4 – p-JNK

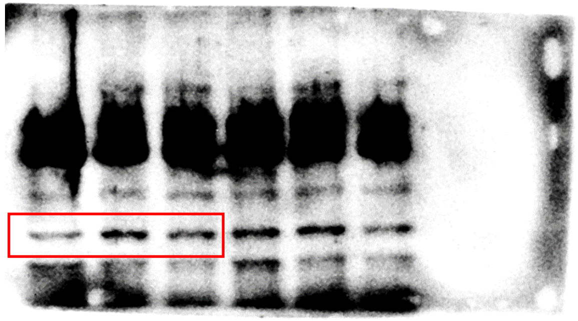

Figure 4 – p38

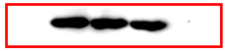

Figure 4 –ERK

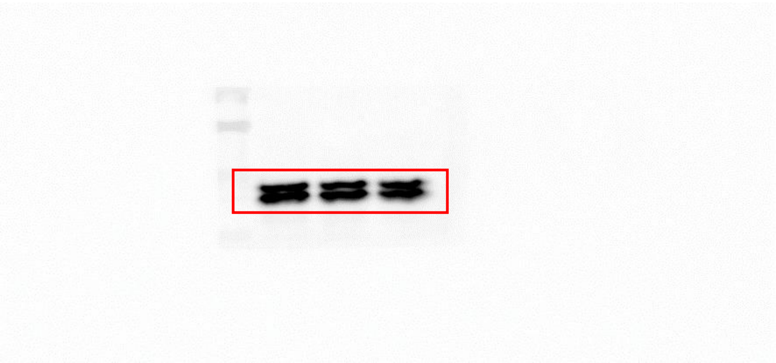

Figure 4 –JNK

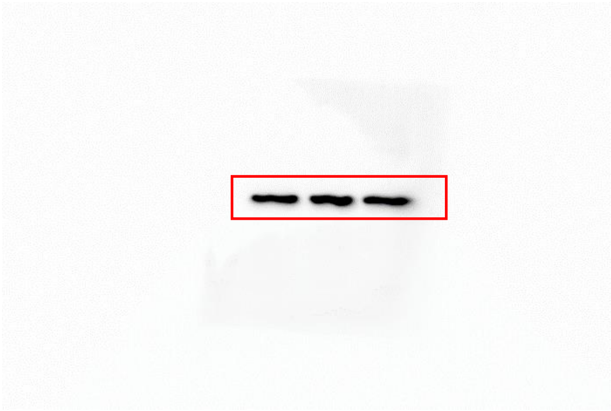

Figure 4 – β-actin

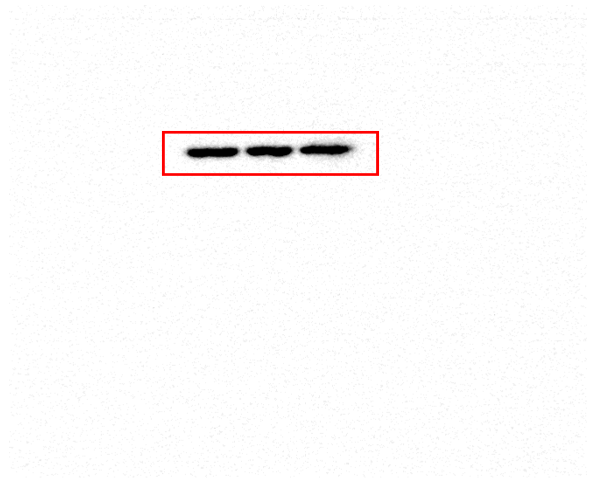

Figure 4 – p-cFos

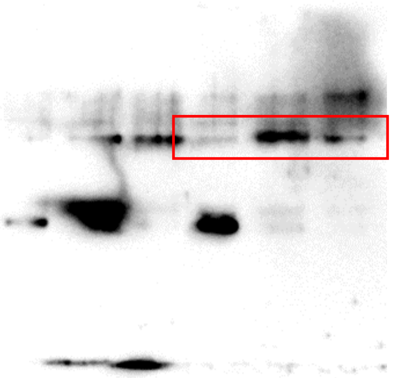

Figure 4 – p-cJun

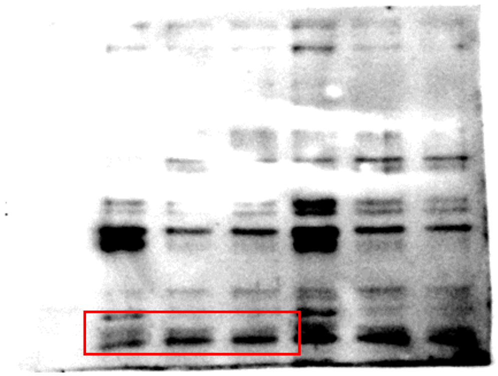

Figure 4 – lamin B1

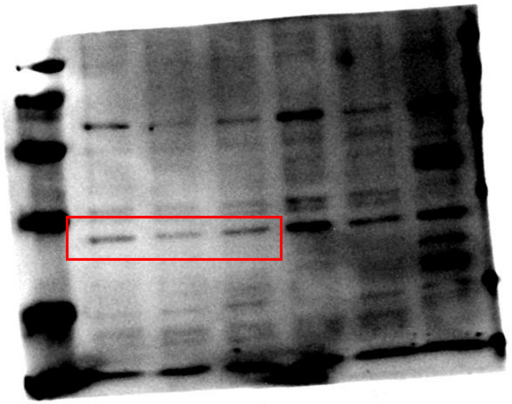

Supplement: Supplementary Materials — Uncropped images of Western blot bands are provided in the supplementary material file. [file 4252917.f1.pdf]
